# Comparing diversity patterns and processes of microbial community assembly in water column and sediment in Lake Wuchang, China

**DOI:** 10.7717/peerj.14592

**Published:** 2023-01-05

**Authors:** Xuemei Li, Zihao Meng, Kang Chen, Feifei Hu, Lu Liu, Tingbing Zhu, Deguo Yang

**Affiliations:** Yangtze River Fisheries Research Institute, Chinese Academy of Fishery Sciences, Wuhan, China

**Keywords:** Microbial community assembly, Species diversity, Functional diversity, Stochastic processes, Environmental factors, Lake Wuchang

## Abstract

The study compare the diversity patterns and processes of microbial community assembly in the water and sediment of Lake Wuchang (China) using high-throughput sequencing of 16S rRNA gene amplicons. A higher microbial α-diversity in the sediment was revealed (*P* < 0.01), and the most common bacterial phyla in water column were Proteobacteria, Cyanobacteria and Actinobacteria, while Proteobacteria, Acidobacteria, Chloroflexi and Nitrospirae were dominant in sediment. Functions related to phototrophy and nitrogen metabolism primarily occurred in the water column and sediment, respectively. The microbial communities in water column from different seasons were divided into three groups, while no such dispersion in sediment based on PCoA and ANOSIM. According to Pearson correlation analysis, water temperature, dissolved oxygen, water depth, total nitrogen, ammonium, and nitrite were key factors in determining microbial community structure in water column, while TN in sediment, conductivity, and organic matter were key factors in sediment. However, the stochastic processes (|βNTI| < 2) dominated community assembly in both the water column and sediment of Lake Wuchang. These data will provide a foundation for microbial development and utilization in lake water column and sediment under the circumstances of increasing tendency of lake ecological fishery in China.

## Introduction

Microbes, as the most abundant, diverse, and functionally important organisms on Earth (Shoemaker, Locey & Lennon, 2017), perform fundamental ecosystem services such as primary production, trophic transfer, nutrient recycling, and waste decomposition in aquatic environments (Fuhrman & Caron, 2015; Worden et al., 2015; Zhao et al., 2017). In lakes, the microbial community composition varies with different environmental forcing features such as seasonality (Gilbert et al., 2012) and natural disturbance (Jones et al., 2008). Recent advances in high-throughput technologies coupled with reduced cost have enabled the application of microbial community analysis for monitoring of microbial quality and diversity in the aquatic environment (Marti et al., 2017; Vadde et al., 2019; Zhang et al., 2021).

Lake water and sediment are different habitats; the former acts as a carrier of biotic and abiotic substances, while the latter serve as sinks or sources for nutrient cycling (Battin et al., 2009). Such differences may account for different microbial communities in lakes. The bacteria from rainfall, groundwater, organisms develop within the lake and soil constitute microbial communities in lake water (Nelson, 2009), and they are expected to strongly differ due to changes in hydrologic conditions, surrounding heterogeneous landscapes, water quality, land uses, and geomorphology (Mari et al., 2014; Tang et al., 2020; Nevers et al., 2020; Zhang et al., 2021). Meanwhile, long-term sediment accumulation, deposition, and erosion shape the microbial communities in sediment developed during sedimentary processes (Guo et al., 2018). To date, a large number of studies have investigated the relative influence of environmental factors on microbial communities in lakes. For example, in the Yellow River, it was found that pH, dissolved organic carbon, and suspended particulate sediment were the main factors controlling the water bacterial community, while the sediment bacterial community was primarily influenced by the pH, nitrate nitrogen, and water content (Xia et al., 2014). In glacier-fed aquatic systems, the bacterial communities were significantly correlated with mean annual precipitation in water but were associated with nitrogen sources and pH in sediment (Zhang et al., 2021).

As central issues in microbial ecology, disentangling the drivers of microbial community structure and function in response to environmental change and understanding the relevant ecological mechanisms are of increasing interest (Zhou & Ning, 2017; Goldford et al., 2018). Currently, it has been accepted that both deterministic and stochastic processes occur simultaneously during the assembly of microbial communities (Hanson et al., 2012; Dini-Andreote et al., 2015). Various theoretical models and practical algorithms quantifying the importance of both deterministic and stochastic processes have been developed and applied, and null models are popular in analysis of microbial community assembly (Chase et al., 2011; Zhou et al., 2014). However, few study on processes of microbial community assembly in lakes connected rivers.

Lake Wuchang is located in the southwest of Anhui Province, China, in the downstream area of the Yangtze River. As it is connected with the Yangtze River, the water level varies depending on the balance between the Yangtze River and Lake Wuchang (Li et al., 2021b). As the largest freshwater lake in the Wanhe river system (a tributary of the Yangtze River in Anhui province), Lake Wuchang provides resources for local fishery development, although multiple stressors from human activities and abiotic factors have been imposed on the lake’s ecosystem. Accordingly, continuous water quality monitoring is essential for protecting this ecosystem. The microbial community as one of the most important bioindicators of water quality and the degree of contamination was monitored in this study by high-throughput sequencing of 16S rRNA gene amplicons on the Illumina platform. The specific objectives were to (i) study the spatial and temporal variation in microbial diversity and abundance in Lake Wuchang water and sediment, (ii) determine the potential functions of bacteria in water and sediment, and (iii) assess the influence of environmental factors on microbial diversity patterns and the community assembly mechanisms based on null models. Therefore, the study makes a fundamental contribution to the mechanistic understanding necessary for a predictive microbial ecology of fishery lakes.

## Materials and Methods

### Sampling and lake Characteristics

**Figure 1:**
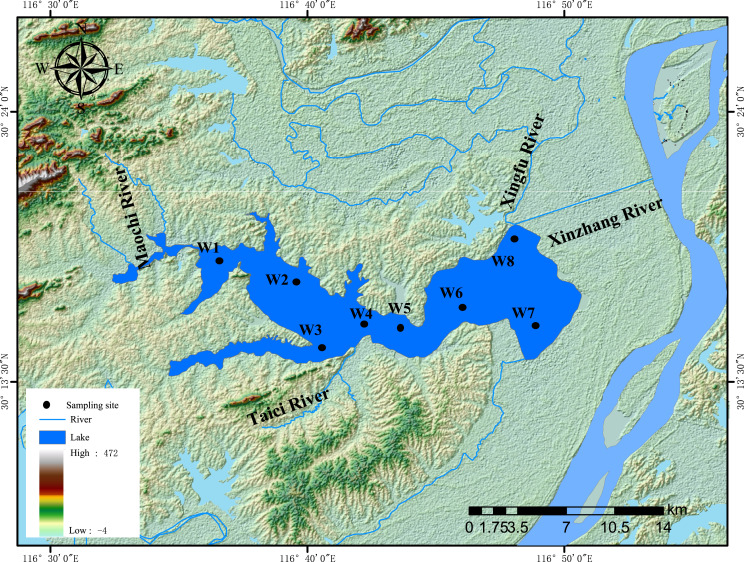
Sampling sites. Location map of the study area and sampling sites in Lake Wuchang, China.

Lake Wuchang (116°36′–116°53′E and 30°14′–30°20′N) is located in the southwest of Anhui Province, on the left bank of the Yangtze River (Fig. 1). It has a surface area of 86 km^2^, a maximum depth of 5 m, and an average depth of 2.5 m. The mean annual temperature and annual precipitation of Lake Wuchang are 16.4 °C and 1,299.6 mm, respectively. The lake water comes from three main rivers: the Taici, Maochi, and Yatan. The lake water outflows downstream through the Xingfu River and Xinzhang River; the latter is connected with the Yangtze River. Thus, backflow occurs annually in May when the water level of Yangtze River is higher, and the water level of Lake Wuchang fluctuates with the Yangtze River water level during the year (Li et al., 2021b).

Water samples were collected from eight monitoring sites (W1–W8) within Lake Wuchang in four seasons (June, August, and October in 2020 and January in 2021). Surface water (top 50 cm) was collected with a 5 L plexiglass water collector in the middle of each month. Subsamples of 1,500 ml water were divided into three parts: 500 mL was used for analyzing Chl-a; 500 mL was used for other water physicochemical parameters, while the remaining 500 mL was used for microbial analysis. Sediment samples were collected from the same water sampling sites. A grab sampler was used to collect sediment samples from the top layer (0–5 cm). For each sampling site, 25 g of sediment was put in a self-sealing bag and transferred to the laboratory, of which 20 g samples were analyzed for sediment physicochemical parameters, and 5 g samples were used for microbial analysis.

### Physicochemical analysis

For collecting Chl-a, the 500 mL water sample was filtered with a cellulose acetate membrane (0.45 μm pore size) and then extracted with hot ethanol (Lorenzen, 1967). The concentration of Chl-a was determined *via* spectrophotometry using a UV-2600PC UV–Vis spectrophotometer (Shimadzu Inc., Kyoto, Japan). The water temperature (WT), dissolved oxygen (DO), conductivity (EC), and pH were measured with a multiparameter water quality analyzer (Hach HQ40D; Hach, Loveland, CO, USA) at the water surface *in situ*. The transparency was measured by a Secchi disk (SD). The water depth (WD) was measured with Saybolt disk and an SM-5 sounder. The concentrations of total nitrogen (TN), total phosphorus (TP), ammonium (NH_4_^+^-N), nitrate (NO_3_^−^-N), nitrite (NO_2_^−^-N), phosphate (PO_4_^−^-P), chemical oxygen demand (COD), and total suspended solid (TSS) were analyzed using a portable multiparameter spectrophotometer (Hach DR1900; Hach, Loveland, CO, USA) according to the manufacturer’s manual. For sediment, the concentrations of organic matter (OC), TN in sediment (STN), and TP in sediment (STP) were analyzed according to the National standard method (Huang, 1999). The physicochemical parameters are shown in Table S1. Differences in environmental variables between different months were determined by one-way ANOVA using the least significant difference (LSD) test at a 5% significance level.

### DNA extraction and Illumina sequencing

Each 500 ml water sample for 16S rRNA gene analysis was filtered with a 0.2 µm pore-size polycarbonate filter (Millipore, Burlington, MA, USA) using a vacuum pump. The filters and 5 g sediment were stored at −20 °C in a vehicle-mounted refrigerator during transportation and subsequently stored at −80 °C in the laboratory until DNA extraction. Microbial DNA in water and sediment was extracted using a Dneasy Powerwater Kit (Qiagen, Hilden, Berlin, Germany) and a PowerSoil DNA Isolation Kit (MOBIO, Berlin, Germany), respectively. The 16S rRNA genes were amplified by polymerase chain reaction (PCR) using the universal primers 515F (5′-GTGCCAGCMGCCGCGG TAA-3′) and 909R (5′-CCCCGYCAATTCM TTTRAGT-3′) targeting the V4–V5 regions as used before (Li et al., 2021a).

The PCR amplification was performed using a touchdown program as described previously (Xiao et al., 2021a; 2021b): 94 °C for 3 min followed by 30 cycles of 94 °C for 40 s, 56 °C for 60 s, 72 °C for 60 s, and a final extension at 72 °C for 10 min until the reaction was halted by the user. The PCR products were separated by 2% agarose gel electrophoresis, and negative controls were performed to ensure there was no contamination. Triplicate PCRs for each sample were conducted and purified using a DNA Gel Extraction Kit (Axygen, Union City, CA, USA). The bar-coded amplicons from each sample were pooled with equimolar concentrations and then were sequenced on an Illumina HiSeq PE250 platform (Illumina Inc., San Diego, CA, USA) by Guangdong Meilikang Bio-science Ltd., China, following the manufacturer’s protocols (Caporaso et al., 2012).

### Data processing and taxonomic assignment

We used QIIME (v1.9.0) to process and quality the raw fastq files according to process described by Caporaso et al. (2010). According to the barcode of each sample, all sequence reads were trimmed and assigned to each sample. High quality sequences without ambiguous base ‘N’ (length > 300 bp and average base quality score > 30) were used for downstream analysis. Then, UCHIME algorithm were used to remove Chimera sequences (Edgar et al., 2011) and UCLUST algorithm was used to cluster the processed sequences with *≥*97% similarity to the same Operational Taxonomic Units (OTUs). Taxonomic assignments of each OTU were determined using the RDP classifier (Wang et al., 2007).

### Microbial diversity and functional annotation

Alpha diversity indices including ACE and Chao1 (species richness estimators), the Shannon index (a combination of richness and evenness), and Simpson diversity were calculated. Statistical differences of α-diversity indices among different seasons in water and sediment of Lake Wuchang were tested using Kruskal–Wallis tests. Beta diversity as PCoA based on weighted UniFrac distance was calculated, and analysis of similarities (ANOSIM) was used to verify the difference between seasonal groups using the vegan package of the R software.

Functional annotation was performed using the package FAPROTAX on the normalized OTU table (Louca, Parfrey & Doebeli, 2016; Louca et al., 2016). FAPROTAX is a manually constructed database that maps prokaryotic taxa (*e.g*., species or genus) to putative functions based on available literature for cultured representatives, with a focus on marine and lake biogeochemistry. In this study, each taxonomically annotated OTU was compared against the FAPROTAX_1.1 database automatically in a Linux system.

### Comparison of taxonomy and function between water and sediment habitats

Taxonomy profiles at the genus level (summarized from OTUs with >0.1% relative abundance) between water and sediment habitats were compared using linear discriminate analysis (LDA) effect size (LEfSe) (Segata et al., 2011). The predicted functional profiles from FAPROTAX annotation on the basis of raw OTU tables were compared between water and sediment habitats. It was defined as significant when a two-sided White’s non-parametric t-test with Benjamini–Hochberg false discovery rate (FDR) yielded a *P*-value <0.05 and an LDA >2.0. The comparisons were visualized on the software STAMP v2.1.3 (Parks et al., 2014).

### Ecological processes governing the microbial community assembly

To reveal the potential controlling factors for the community composition, we used a correlation heatmap to reveal the driving environmental factors for dominant genera using the corrplot package for the R environment. To assess the relative importance of deterministic and stochastic processes driving microbial community assembly, a null model analysis was conducted following the framework described by Stegen et al. (2013) and Ning et al. (2020). The phylogenetic β-diversity was quantified using beta mean nearest taxon distance (βMNTD) and beta nearest taxon index (βNTI) *via* the ‘picante’ package for the R environment. The βMNTD represents the phylogenetic distance between each OTU in one community and its closest relative in a second community, and βNTI quantifies the difference between observed βMNTD and the null distribution of βMNTD. A value of |βNTI| < 2 indicates that the community composition is the result of stochastic processes, While |βNTI| > 2 suggests that the community assembly is governed primarily by deterministic processes (Sun et al., 2021). The significance of differences of the actual communities from those of the related null expectation is based on Wilcoxon tests.

## Results

### Microbial diversity, taxonomy, and community structure

**Figure 2:**
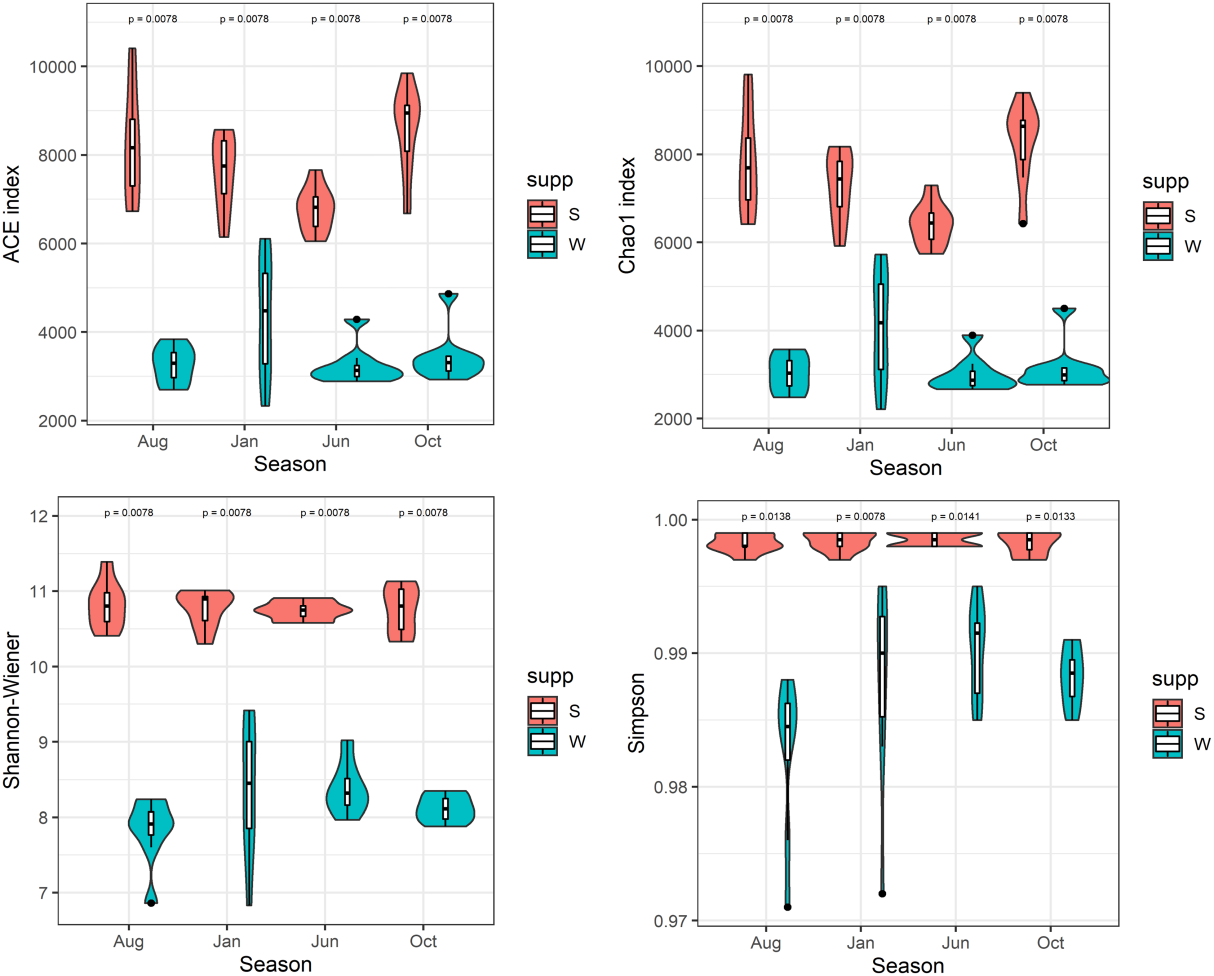
α-diversity indices. Comparison of α-diversity indices of microbial communities in the water column and sediment. W and S represent the water column and sediment, respectively.

After quality filtering and normalization, totals of 1,036,257 and 1,951,966 high-quality reads (average length = 420 bp) were generated from 32 water and 32 sediment samples in Lake Wuchang, averaging 32,383 and 60,998 reads per sample, respectively. The microbial α-diversity indices among different seasons in water and sediment habitats were compared. The richness indices ACE and Chao1 (means = 7,798 and 7,742, respectively) and the Shannon and Simpson diversity indices (means = 10.8 and 0.99, respectively) in the sediment were significantly higher than in water (means = 3,577, 3,522, 8.2, and 0.98, respectively; *P* < 0.01) (Fig. 2).

**Figure 3:**
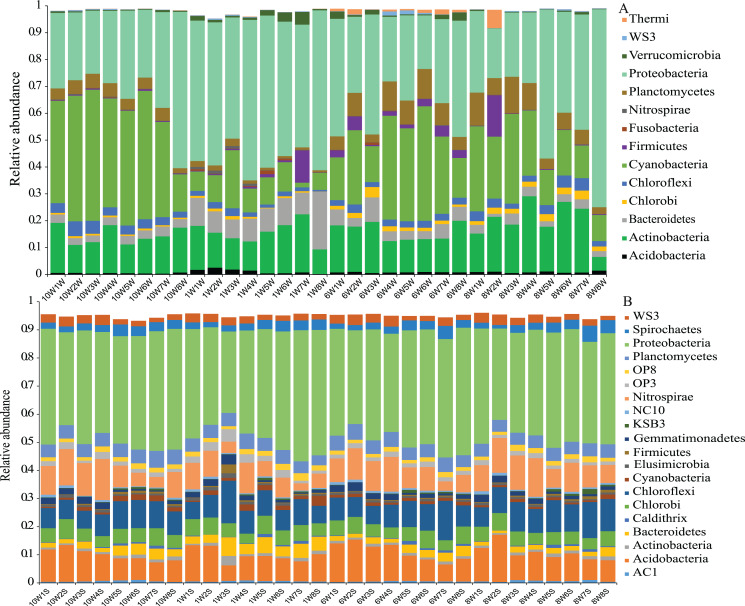
Microbial community composition. Phylum-level distribution of microbial communities from each sampling site in the water column (A) and sediment (B) in Lake Wuchang. The numbers 10, 1, 6, and 8 represent different months; W1–W8 represent sampling sites; and the letters W and S represent the water column and sediment, respectively.

The number of OTUs was analyzed for each sample using a 97% sequence similarity cutoff value. A total of 28,727 OTUs were detected in water and sediment, and these were classified and grouped under 14 and 21 phylum-level taxonomic groups in water and sediment, respectively (Fig. 3). In the water column, the most common phyla of bacteria were Proteobacteria (average 39.19%), Cyanobacteria (24.12%), Actinobacteria (15.73%), Planctomycetes (5.18%), Firmicutes (1.74%), Bacteroidetes (5.24%), Chloroflexi (2.75%), and Chlorobi (1.29%); the dominant bacterial phylum in the sediment was also Proteobacteria (38.90%), followed by Acidobacteria (9.84%), Chloroflexi (8.93%), Nitrospirae (8.61%), Chlorobi (5.07%), Planctomycetes (4.76%), Spirochaetes (2.97%), Bacteroidetes (2.80%), WS3 (2.59%), Gemmatimonadetes (1.58%), and Cyanobacteria (1.22%).

**Figure 4:**
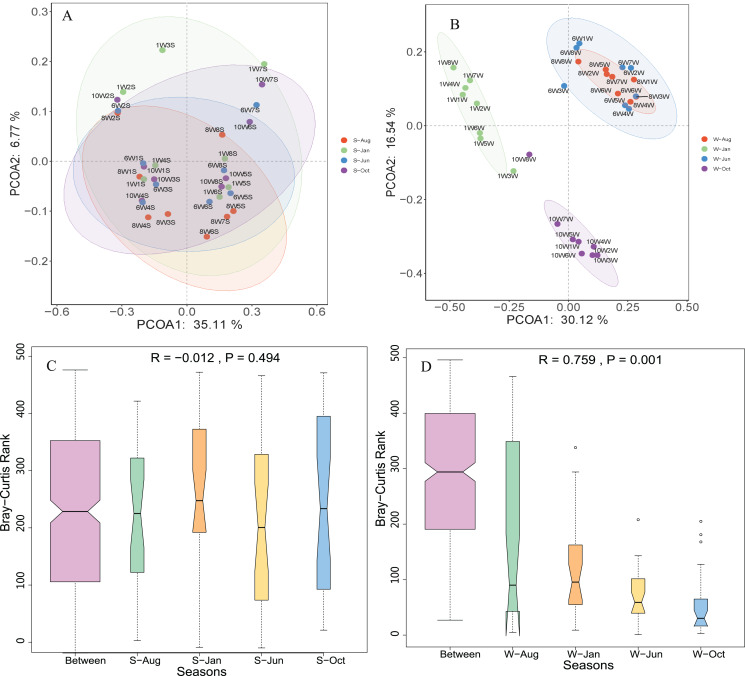
Microbial community β-diversity visualized using PCoA ordination (A and B) with Bray–Curtis distance and ANOSIM tests (C and D) between different seasons in the water column and sediment. The numbers 10, 1, 6, and 8 represent different months; W1–W8 represent sampling sites; and the letters W and S represent the water column and sediment, respectively.

Unconstrained principal coordinates analysis (PCoA) of the Bray–Curtis distance was performed to evaluate β-diversity among different sampling sites (Fig. 4). The first two axes explained 46.7% and 41.9% of the microbial community variation in water and sediment, respectively. For water samples, the microbial communities were divided into three groups according to the seasons: samples collected in October clustered together, while samples collected in January clustered as another group, and samples collected in June and August clustered together as a third group (Fig. 4A). Samples collected from different seasons in sediment revealed no dispersion (Fig. 4B). According to ANOSIM, the differences between seasonal groups in water were greater than between groups in sediment (R = 0.759, *P* = 0.001 and R = −0.012, *P* = 0.488, respectively, Figs. 4C and 4D).

### Microbial community and predicted function between Water and Sediment habitats

**Figure 5:**
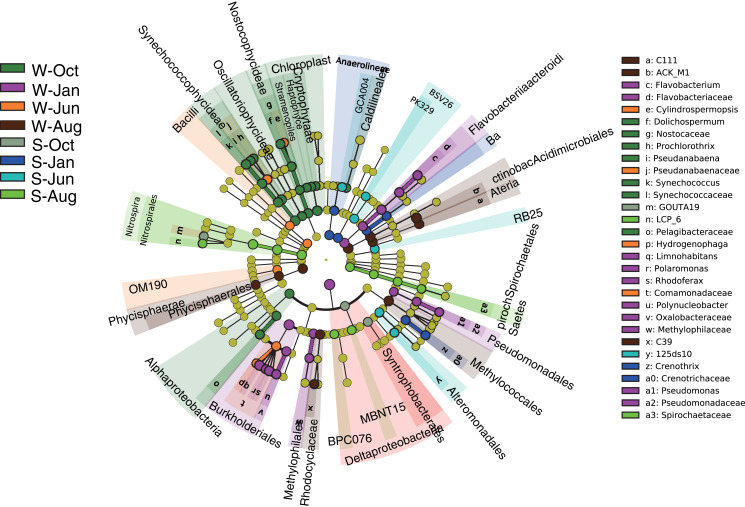
LEfSe results. LEfSe results showing the taxonomic differences of microbial communities between different seasons in the water column and sediment. Different colors represent different seasons, whereas the yellow circles represent the taxa with nonsignificant differences. Statistical analyses were performed using log linear discriminant analysis (LDA) with LDA > 2.0 and *P* < 0.05 after correction by the Benjamini and Hochberg false discovery rate (FDR) test. W and S represent the water column and sediment, respectively.

Linear discriminant effect size (LEfSe) analysis was implemented to designate the specialized microbial lineages for different habitats. A total of 30 microbial taxa were found to be significantly different between different seasons in the water column and sediment (Fig. 5). Generally, the mean proportions of Actinobacteria (phylum Actinobacteria) and Flavobacteriaceae (phylum Bacteroidetes), Synechococcophycideae, Chloroplast and Nostocophycideae (phylum Cyanobacteria), Hydrogenophaga (phylum Proteobacteria), and a group of unassigned microbes were significantly higher in the water column, while Nitrospirales (phylum Nitrospirae) were enriched in sediment.

**Figure 6:**
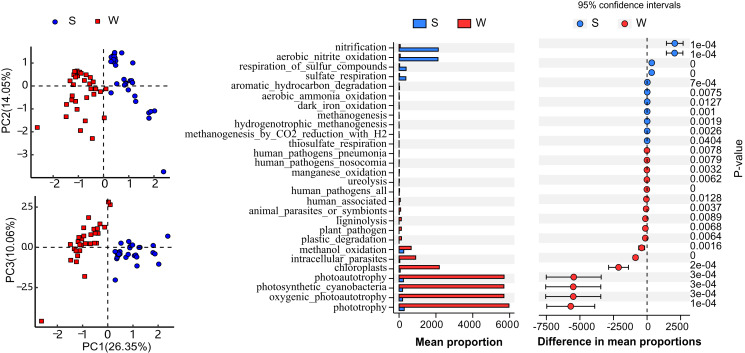
Function diversity. Putative function profiles of microbial communities in the water column and sediment. (A) PCA plots comparing the entire function profiles. (B) Functional categories differing significantly between the water column and sediment. W and S represent the water column and sediment, respectively.

Functional annotation of OTUs revealed a rich repertoire of metabolic functional groups in the water and sediment microbial communities. There were 73 annotated functional groups in water and sediment habitats, indicating high functional diversity in Lake Wuchang comparing to other lakes and rivers (Tang et al., 2020). Principal component analysis (PCA) of functional profiles showed distinct separation between water and sediment habitats, and the functional variation in the water column was much higher than that in sediment (Fig. 6A). Among the putative functions, chemoheterotrophy (contributed by the phyla Acidobacteria, Proteobacteria, and Verrucomicrobi), methanotrophy (contributed by the phylum Proteobacteria), aerobic chemoheterotrophy (contributed by the phyla Acidobacteria, Proteobacteria, Firmicutes, and Bacteroidetes), photoautotrophy (contributed by the phyla Cyanobacteria, Chlorobi, and Proteobacteria), phototrophy (contributed by the phyla Chlorobi, Cyanobacteria, and Proteobacteria), and nitrification (contributed by the phyla Nitrospirae and Proteobacteria) were the most abundant groups in both habitats. By using White’s non-parametric t-test, the functional groups of phototrophy, photoautotrophy, oxygenic photoautotrophy, photosynthetic cyanobacteria chloroplasts, and intracellular parasites were significantly enriched in the water column, while the mean proportions of nitrification, aerobic nitrite oxidation, respiration of sulfur compounds, and sulfate respiration were higher in sediment (Fig. 6B).

### Influential factors and stochastic processes in microbial community assembly

**Figure 7:**
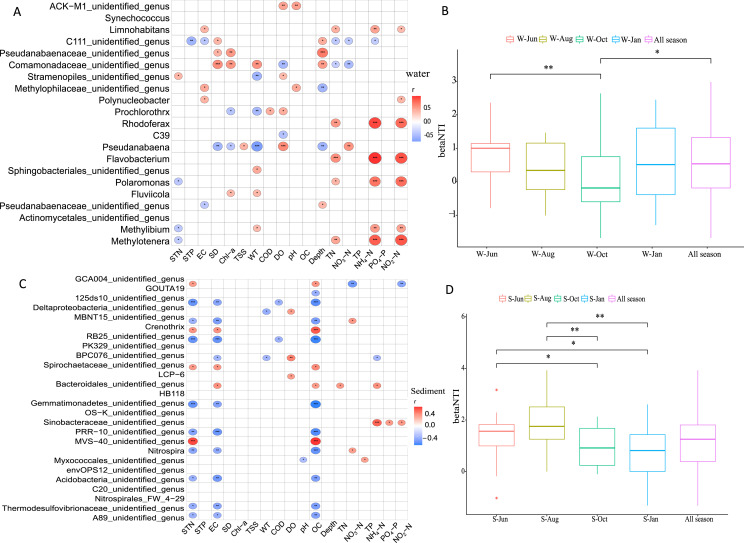
Drivers factors of microbial community composition. Drivers of microbial community composition based on a correlation heatmap in the water column (A) and sediment (C) and the relative importance of stochastic mechanisms in community assembly in the two habitats (B, D). STN, TN in sediment; STP, TP in sediment; EC, conductivity; SD, Secchi disk transparency; Chl-a, chlorophyll a concentration; TSS, total suspended solid; WT, water temperature; COD, chemical oxygen demand; DO, dissolved oxygen; OC, organic matter; WD, water depth; TN, total nitrogen; TP, total phosphorus; NH_4_^+^-N, ammonium; NO_3_^−^-N, nitrate; NO_2_^−^-N, nitrite; and PO_4_^−^-P, phosphate. W and S represent water column and sediment, respectively. *P < 0.05, **P < 0.01, ***P < 0.001.

Pearson correlation analysis was employed to elucidate the driving environmental factors of dominant genera in different seasons in the water column and sediment. It was revealed that the variation in microbial communities was related to six environmental variables in the water column (Fig. 7A) and three environmental variables in sediment (Fig. 7C). In general, WT, DO, WD, TN, NH_4_^+^-N, and NO_2_^−^-N were the most important factors, with generally positively correlations in structuring the microbial community assemblages in the water column, while STN, EC, and OC were the most important factors in sediment, each with negative correlations.

To disentangle the relative importance of stochastic mechanisms from deterministic mechanisms in shaping the microbial community structure, βNTI was calculated for paired samples. The βNTI values for the microbial community in the water column and sediment ranged from −1 to +1 and 0 to +2, respectively (Figs. 7B and 7D). The relative frequency of βNTI suggested that stochastic processes may have played more important roles in shaping the lake microbiome.

## Discussion

In this study, the microbial α-diversity in the sediment was much higher than that in the water column in all seasons in Lake Wuchang, a result that was consistent with previous findings in lakes, estuaries, and springs (Feng et al., 2009; Cole et al., 2013; Zhang et al., 2021). It is well recognized that sediments normally contain highly diverse microbes that act as a reservoir from which recruitment and subsequent deposit of microbes from surrounding areas (*e.g*., soils, sands, and plant debris) occur (Crump, Amaral-Zettler & Kling, 2012). Moreover, sediments share some properties with soils such as long-term deposition and erosion, processes that increase nutrient contents and moisture and potentially alter the pH, finally increasing microbial biomass and diversity (Müller-Nedebock, Chivenge & Chaplot, 2016; Du et al., 2020). These processes may potentially explain the relationship between sediment bacterial diversity and sediment properties, *i.e*., nitrogen and organic matter (STN and OC) (Fig. 7).

The microbial communities in this study showed a clear separation between the metrics (Figs. 2 and 3), indicating that the habitat type (water and sediment) was the main reason for the differences in microbial community composition. In Lake Wuchang, Proteobacteria, Cyanobacteria, and Actinobacteria were dominant in the water column, while Proteobacteria, Acidobacteria, Chloroflexi, Nitrospirae, Chlorobi, and Planctomycetes dominated in the sediment, patterns that were also supported by the LEfSe analysis (Fig. 5). Proteobacteria, which is typical of freshwater ecosystems (Tamames et al., 2010), is also commonly the most abundant phylum in sediment or soil; the members of the phylum play a role in degradation and metabolism in lake sediments (Bai et al., 2012; Huang et al., 2017). According to the putative functions in this study, phylum Proteobacteria was involved in the processes of chemoheterotrophy, methanotrophy, aerobic chemoheterotrophy, photoautotrophy, phototrophy, and nitrification (Fig. 6). Actinobacteria are widely distributed in aquatic ecosystems, and they play important roles in decomposition of complex materials from dead fish and algae and in recycling of the nutrients resulting in humus formation (Sharma, 2014). Cyanobacteria as the oxygenic photosynthetic bacteria play a significant role in the nitrogen cycle as well as in the cycles of oxygen and carbon (Tomitani et al., 2006). In sediment, Acidobacteria are reported to be one of the most abundant phyla, having an important role in biogeochemical cycles of organic matter decomposition and nutrient cycling (Catão et al., 2014). Chloroflexi are photoautotrophic microbes that possibly participate in the degradation of organic compounds in sediment, while Nitrospirae are strong indicators of the nitrogen cycle and can potentially serve as a warning for the occurrence of algal blooms in lakes (Ligi et al., 2014). Thus, the present study collectively contributes to a more comprehensive overview of the biogeochemical cycling mediated by microbial communities and is benefit to environmental protection in lakes.

In addition to microbial diversity, higher functional diversity in the water column compared with the sediment was observed (Fig. 6). According to the functional annotation of OTUs, significant enrichment of genes associated with phototrophy and photoautotrophy in the water column and nitrification and aerobic nitrite oxidation in the sediment were found. This may be due to the different dominant bacterial phyla (Cyanobacteria and Nitrospirae) in the two habitats. They were consistent with previous research, suggesting that sediment plays crucial roles in denitrification and nitrogen uptake as well as export of nitrogen to lake water (Racchetti et al., 2017). Future efforts should be taken into consideration to investigate the phyla Cyanobacteria and Nitrospirae and their function profiles of a broader range lakes with different trophic status.

According to the PCoA plot, the microbial communities from different seasons were divided into three groups for water samples, while samples collected from sediment revealed no dispersion (Fig. 5), suggesting that the microbial communities in sediment were less affected by seasonal environmental variations compared with those in the water column. This reason may be due to the environmental factors having changed more significantly in different seasons in the water column (Xu et al., 2019), a significant variation of environmental factors between different seasons was revealed in the present study (Table S1). In addition, WT, DO, WD, TN, NH_4_^+^-N, and NO_2_^−^-N were key factors for bacterial community structure in the water column, while STN, EC, and OC were key factors in sediment. This demonstrated that nitrogen compounds were a major shaper of microbial communities both in the water column and sediment in Lake Wuchang, which is consistent with previous studies (Huang et al., 2017). WT and DO, as important factors affecting bacterial growth and decomposition, were found to be related to microbial communities among Lake Bosten catchment (Tang et al., 2020). Mladenov et al. (2011) reported that lake bacteria were influenced by the OC and that they produced chromophoric organic compounds with major implications for carbon cycling.

Our datas clearly support the prominent role of stochastic processes in shaping the microbial community assembly in both the water column and sediment in Lake Wuchang, although environmental factors also played important roles. Moreover, microbial community assembly had clear seasonal succession patterns in sediment (Fig. 7). Many studies have reported that stochastic processes dominated microbial or microeukaryotic community assembly in rivers and lakes (Chase, 2010; Caruso et al., 2011; Yang et al., 2016; Tang et al., 2020). The environmental perturbations may account for this mechanisms in the Lake Wuchang, as it is connected with the Yangtze River; the water level fluctuates, and water mixing increases nutrient input to stimulate microbial diversity and increase the mutation rate (Zhou et al., 2014). However, to fully understand the microbial community assembly mechanisms in lakes, future microbial community ecology research in different lakes should focus on the effects of sampling scale (different altitudes), species interactions, and other possible stochastic factors.

## Conclusions

Through an entire year survey spanning four seasons, significant differences in both microbial а-diversity and functional diversity between the water column and sediment have been found in Lake Wuchang. Here we showed that a dramatic lower in the microbial diversity of the water column and in functions related to phototrophy and nitrogen metabolism primarily occurred in the water column and sediment, respectively. In addition, the stochastic processes dominated community assembly in both water and sediment habitats, although environmental factors were also important. This study represents one attempt to recoverthe microbial diversity patterns and the community assembly mechanisms in fishery lake ecosystems. Species interactions in microbial community ecology in different scales lakes should be considered in the future.

## Supplemental Information

10.7717/peerj.14592/supp-1 Supplemental Information 1 Seasonal changes of water environmental factors in Lake Wuchang (mean ± SD).

10.7717/peerj.14592/supp-2 Supplemental Information 2 Environmental factors raw data.

## Additional Information and Declarations

### Data Availability

The following information was supplied regarding data availability: The original sequences are available at the Sequence Read Archive (SRA) in the National Center for Biotechnology Information (NCBI): PRJNA791709.

## References

[ref-1] Bai Y, Shi Q, Wen D, Li Z, Jefferson WA, Feng C, Tang X (2012). Bacterial communities in the sediments of Dianchi Lake, a partitioned eutrophic waterbody in China. PLOS ONE.

[ref-2] Battin TJ, Luyssaert S, Kaplan LA, Aufdenkampe AK, Richter A, Tranvik LJ (2009). The boundless carbon cycle. Nature Geoscience.

[ref-3] Caporaso JG, Kuczynski J, Stombaugh J, Bittinger K, Bushman FD, Costello EK, Fierer N, Peña AG, Goodrich JK, Gordon JI, Huttley GA, Kelley ST, Knights D, Koenig JE, Ley RE, Lozupone CA, McDonald D, Muegge BD, Pirrung M, Reeder J, Sevinsky JR, Turnbaugh PJ, Walters WA, Widmann J, Yatsunenko T, Zaneveld J, Knight R (2010). QIIME allows analysis of high-throughput community sequencing data. Nature Methods.

[ref-4] Caporaso JG, Lauber CL, Walters WA, Berg-Lyons D, Huntley J, Fierer N, Owens SM, Betley J, Fraser L, Bauer M, Gormley N, Gilbert JA, Smith G, Knight R (2012). Ultra-high-throughput microbial community analysis on the Illumina HiSeq and MiSeq platforms. ISME Journal.

[ref-5] Caruso T, Chan Y, Lacap DC, Lau MCY, McKay CP, Pointing SB (2011). Stochastic and deterministic processes interact in the assembly of desert microbial communities on a global scale. The ISME Journal.

[ref-6] Catão EC, Lopes FA, Araújo JF, de Castro AP, Barreto CC, Bustamante MMC, Quirino BF, Krüger RH (2014). Soil acidobacterial 16S rRNA gene sequences reveal subgroup level differences between Savanna-Like cerrado and atlantic forest Brazilian biomes. International Journal of Microbiology.

[ref-7] Chase JM (2010). Stochastic community assembly causes higher biodiversity in more productive environments. Science.

[ref-8] Chase JM, Kraft NJB, Smith KG, Vellend M, Inouye BD (2011). Using null models to disentangle variation in community dissimilarity from variation in alphadiversity. Ecosphere.

[ref-9] Cole JK, Peacock JP, Dodsworth JA, Williams AJ, Thompson DB, Dong H, Wu G, Hedlund BP (2013). Sediment microbial communities in Great Boiling Spring are controlled by temperature and distinct from water communities. The ISME Journal.

[ref-10] Crump BC, Amaral-Zettler LA, Kling GW (2012). Microbial diversity in arctic freshwaters is structured by inoculation of microbes from soils. The ISME Journal.

[ref-11] Dini-Andreote F, Stegen JC, van Elsas JD, Salles JF (2015). Disentangling mechanisms that mediate the balance between stochastic and deterministic processes in microbial succession. Proceedings of the National Academy of Sciences of the United States of America.

[ref-12] Du L, Wang R, Gao X, Hu Y, Guo S (2020). Divergent responses of soil bacterial communities in erosion-deposition plots on the Loess Plateau. Geoderma.

[ref-13] Edgar RC, Haas BJ, Clemente JC, Quince C, Knight R (2011). UCHIME improves sensitivity and speed of chimera detection. Bioinformatics.

[ref-14] Feng B-W, Li X-R, Wang J-H, Hu Z-Y, Meng H, Xiang L-Y, Quan Z-X (2009). Bacterial diversity of water and sediment in the Changjiang estuary and coastal area of the East China Sea. FEMS Microbiology Ecology.

[ref-15] Fuhrman JA, Caron DA, Yates MV, Nakatsu CH, Miller RV, Pillai SD (2015). Heterotrophic planktonic microbes: virus, bacteria, archaea, and protozoa. Manual of Environmental Microbiology.

[ref-16] Gilbert JA, Steele JA, Caporaso JG, Steinbrück L, Reeder J, Temperton B, Huse S, McHardy AC, Knight R, Joint I, Somerfield P, Fuhrman JA, Field D (2012). Defining seasonal marine microbial community dynamics. The ISME Journal.

[ref-17] Goldford JE, Lu N, Bajić D, Estrela S, Tikhonov M, Sanchez-Gorostiaga A, Segrè D, Mehta P, Sanchez A (2018). Emergent simplicity in microbial community assembly. Science.

[ref-18] Guo X-P, Lu D-P, Niu Z-S, Feng J-N, Chen Y-R, Tou F-Y, Liu M, Yang Y (2018). Bacterial community structure in response to environmental impacts in the intertidal sediments along the Yangtze Estuary, China. Marine Pollution Bulletin.

[ref-19] Hanson CA, Fuhrman JA, Horner-Devine MC, Martiny JBH (2012). Beyond biogeographic patterns: processes shaping the microbial landscape. Nature Reviews Microbiology.

[ref-20] Huang XF (1999). Survey, observation and analysis of lake ecology.

[ref-21] Huang W, Chen X, Jiang X, Zheng B (2017). Characterization of sediment bacterial communities in plain lakes with different trophic statuses. MicrobiologyOpen.

[ref-22] Jones SE, Chiu C-Y, Kratz TK, Wu J-T, Shade A, McMahon KD (2008). Typhoons initiate predictable change in aquatic bacterial communities. Limnology and Oceanography.

[ref-23] Li X, Liu L, Zhu Y, Zhu T, Wu X, Yang D (2021a). Microbial community structure and its driving environmental factors in black carp (*Mylopharyngodon piceus*) aquaculture pond. Water.

[ref-24] Li XM, Meng ZH, Hu FF, Liu L, Gong SS, Zhu YJ, Yang DG (2021b). Characteristics of phytoplankton primary productivity in wet and dry seasons and its correlations with environmental factors in Lake Wuchang, Anhui province. Freshwater Fisheries.

[ref-25] Ligi T, Oopkaup K, Truu M, Preema J-K, Nõlvaka H, Mitsch WJ, Mandera Ü, Truua J (2014). Characterization of bacterial communities in soil and sediment of a created riverine wetland complex using high-throughput 16S rRNA amplicon sequencing. Ecological Engineering.

[ref-26] Lorenzen CJ (1967). Determination of chlorophyll and pheo-pigments: spectropho tometric equations. Limnology and Oceanography.

[ref-27] Louca S, Jacques SMS, Pires APF, Leal JS, Srivastava DS, Parfrey LW, Farjalla VF, Doebeli M (2016). High taxonomic variability despite stable functional structure across microbial communities. Nature Ecology and Evolution.

[ref-28] Louca S, Parfrey LW, Doebeli M (2016). Decoupling function and taxonomy in the global ocean microbiome. Science.

[ref-29] Mari L, Casagrandi R, Bertuzzo E, Rinaldo A, Gatto M, Jordán F (2014). Metapopulation persistence and species spread in river networks. Ecology Letters.

[ref-30] Marti R, Ribun S, Aubin J-B, Colinon C, Petit S, Marjolet L, Gourmelon M, Schmitt L, Breil P, Cottet M, Cournoyer B (2017). Human-driven microbiological contamination of benthic and hyporheic sediments of an intermittent peri-urban River assessed from MST and 16S rRNA genetic structure analyses. Frontiers in Microbiology.

[ref-31] Mladenov N, Sommaruga R, Morales-Baquero R, Laurion I, Camarero L, Diéguez MC, Camacho A, Delgado A, Torres O, Chen Z, Felip M, Reche I (2011). Dust inputs and bacteria influence dissolved organic matter in clear alpine lakes. Nature Communications.

[ref-32] Müller-Nedebock D, Chivenge P, Chaplot V (2016). Selective organic carbon losses from soils by sheet erosion and main controls. Earth Surface Processes and Landforms.

[ref-33] Nelson CE (2009). Phenology of high-elevation pelagic bacteria: the roles of meteorologic variability, catchment inputs and thermal stratification in structuring communities. ISME Journal.

[ref-34] Nevers MB, Byappanahalli MN, Nakatsu CH, Kinzelman JL, Phanikumar MS, Shively DA, Spoljaric AM (2020). Interaction of bacterial communities and indicators of water quality in shoreline sand, sediment, and water of Lake Michigan. Water Research.

[ref-35] Ning D, Yuan M, Wu L, Zhang Y, Guo X, Zhou X, Yang Y, Arkin AP, Firestone MK, Zhou J (2020). A quantitative framework reveals ecological drivers of grassland microbial community assembly in response to warming. Nature Communications.

[ref-36] Parks DH, Tyson GW, Hugenholtz P, Beiko RG (2014). STAMP: statistical analysis of taxonomic and functional profiles. Bioinformatics.

[ref-37] Racchetti E, Longhi D, Ribaudo C, Soana E, Bartoli M (2017). Nitrogen uptake and coupled nitrification–denitrification in riverine sediments with benthic microalgae and rooted macrophytes. Aquatic Sciences.

[ref-38] Segata N, Izard J, Waldron L, Gevers D, Miropolsky L, Garrett WS, Huttenhower C (2011). Metagenomic biomarker discovery and explanation. Genome Biology.

[ref-39] Sharma M (2014). Actinomycetes: source, identification, and their applications. International Journal of Current Microbiology and Applied Sciences.

[ref-40] Shoemaker WR, Locey KJ, Lennon JT (2017). A macroecological theory of microbial biodiversity. Nature Ecology & Evolution.

[ref-41] Stegen JC, Lin X, Fredrickson JK, Chen X, Kennedy DW, Murray CJ, Rockhold ML, Konopka A (2013). Quantifying community assembly processes and identifying features that impose them. The ISME Journal.

[ref-42] Sun Y, Zhang M, Duan C, Cao N, Jia W, Zhao Z, Ding C, Huang Y, Wang J (2021). Contribution of stochastic processes to the microbial community assembly on field-collected microplastics. Environmental Microbiology.

[ref-43] Tamames J, Abellan JJ, Pignatelli M, Camacho A, Moya A (2010). Environmental distribution of prokaryotic taxa. BMC Microbiology.

[ref-44] Tang X, Xie G, Shao K, Hu Y, Cai J, Bai C, Gong Y, Gao G (2020). Contrast diversity patterns and processes of microbial community assembly in a river-lake continuum across a catchment scale in northwestern China. Environmental Microbiome.

[ref-45] Tomitani A, Knoll AH, Cavanaugh CM, Ohno T (2006). The evolutionary diversification of cyanobacteria: molecular-phylogenetic and paleontological perspectives. Proceedings of the National Academy of Sciences of the United States of America.

[ref-46] Vadde KK, Feng Q, Wang J, McCarthy AJ, Sekar R (2019). Next-generation sequencing reveals fecal contamination and potentially pathogenic bacteria in a major inflow river of Taihu Lake. Environmental Pollution.

[ref-47] Wang Q, Garrity GM, Tiedje JM, Cole JR (2007). Naïve Bayesian classifier for rapid assignment of rRNA sequences into the new bacterial taxonomy. Applied and Environmental Microbiology.

[ref-48] Worden AZ, Follows MJ, Giovannoni SJ, Wilken S, Zimmerman AE, Keeling PJ (2015). Rethinking the marine carbon cycle: factoring in the multifarious lifestyles of microbes. Science.

[ref-49] Xia N, Xia X, Liu T, Hu L, Zhu B, Zhang X, Dong J (2014). Characteristics of bacterial community in the water and surface sediment of the Yellow River, China, the largest turbid river in the world. Journal of Soils and Sediments.

[ref-50] Xiao F, Liao L, Xu Q, He Z, Xiao T, Wang J, Huang J, Yu Y, Wu B, Yan Q (2021b). Host-microbiota interactions and responses to grass carp reovirus infection in *Ctenopharyngodon idellus*. Environmental Microbiology.

[ref-51] Xiao F, Zhu W, Yu Y, He Z, Wu B, Wang C, Shu L, Li X, Yin H, Wang J, Juneau P, Zheng X, Wu Y, Li J, Chen X, Hou D, Huang Z, He J, Xu G, Xie L, Huang J, Yan Q (2021a). Host development overwhelms environmental dispersal in governing the ecological succession of zebrafish gut microbiota. NPJ Biofilms Microbiomes.

[ref-52] Xu G, Li P, Lu K, Tantaia Z, Zhang J, Ren Z, Wang X, Yu K, Shi P, Cheng Y (2019). Seasonal changes in water quality and its main influencing factors in the Dan River basin. Catena.

[ref-53] Yang J, Ma L, Jiang HC, Wu G, Dong H (2016). Salinity shapes microbial diversity and community structure in surface sediments of the Qinghai-Tibetan Lakes. Scientific Reports.

[ref-54] Zhang L, Delgado-Baquerizo M, Shi Y, Liu X, Yang Y, Chu H (2021). Co-existing water and sediment bacteria are driven by contrasting environmental factors across glacier-fed aquatic systems. Water Research.

[ref-55] Zhao D, Cao X, Huang R, Zeng J, Shen F, Xu H, Wang S, He X, Yu Z (2017). The heterogeneity of composition and assembly processes of the microbial community between different nutrient loading lake zones in Taihu Lake. Applied Microbiology and Biotechnology.

[ref-56] Zhou J, Deng Y, Zhang P, Xue K, Liang Y, Van Nostrand JD, Yang Y, He Z, Wu L, Stahl DA, Hazen TC, Tiedje JM, Arkin AP (2014). Stochasticity, succession, and environmental perturbations in a fluidic ecosystem. Proceedings of the National Academy of Sciences of the United States of America.

[ref-57] Zhou J, Ning D (2017). Stochastic community assembly: does it matter in microbial ecology?. Microbiology and Molecular Biology Reviews.

