# Supplementary material for: Comparing diversity patterns and processes of microbial community assembly in water column and sediment in Lake Wuchang, China
Source: PeerJ. 2023 Jan 5;11:e14592. doi: 10.7717/peerj.14592 (PMC9826614; doi:10.7717/peerj.14592)
Supplement: Supplemental Information 1 [file peerj-11-14592-s001.doc]

## Supplementary Material

**Additional file 1:**

**Table S1**. Seasonal changes of water environmental factors in Lake Wuchang (mean ± SD) Different lowercase letters indicate significant differences between seasonal values

| Environmental factors | June | August | October | January | *P*-value |
| --- | --- | --- | --- | --- | --- |
| WD (m) | 1.79 ± 0.48^c^ | 4.64 ± 0.88^a^ | 2.85 ± 0.27^b^ | 1.39 ± 0.20^c^ | < 0.01 |
| WT (°C) | 24.30 ± 0.73^b^ | 29.89 ± 0.38^a^ | 18.80 ± 0.26^c^ | 8.21 ± 1.92^d^ | < 0.01 |
| DO (mg/L) | 8.08 ± 2.56^b^ | 5.56 ± 2.04^c^ | 8.69 ± 2.77^b^ | 11.08 ± 1.18^a^ | < 0.01 |
| pH | 8.23 ± 0.67^a^ | 7.80 ± 0.21^b^ | 8.34 ± 0.28^a^ | 8.26 ± 0.13^a^ | < 0.05 |
| TSS (mg/L) | 8.50 ± 3.21^b^ | 8.25 ± 1.75^b^ | 8.88 ± 1.73^b^ | 63.12 ± 64.72 ^a^ | < 0.01 |
| SD (cm) | 61.38 ± 23.21^b^ | 93.13 ± 14.38^a^ | 98.75 ± 26.02^a^ | 37.63 ± 18.70^c^ | < 0.01 |
| EC (μs/cm) | 142.61 ± 21.02 | 120.60 ± 10.90 | 127.51 ± 27.31 | 156.08 ± 38.05 | 0.051 |
| TP (mg/L) | 0.11 ± 0.04^ab^ | 0.14 ± 0.04^a^ | 0.09 ± 0.01^b^ | 0.14 ± 0.05^a^ | < 0.01 |
| PO_4_^3−^-P (mg/L) | 0.06 ± 0.03 | 0.08 ± 0.01 | 0.03 ± 0.01 | 0.06 ± 0.05 | 0.472 |
| TN (mg/L) | 1.66 ± 1.21^ab^ | 0.76 ± 1.04^bc^ | 0.34 ± 0.21^c^ | 1.79 ± 0.79^a^ | < 0.01 |
| NH_4_^+^-N (mg/L) | 0.53 ± 0.73^a^ | 0.05 ± 0.05^b^ | 0.01 ± 0.0^b^ | 0.11 ± 0.09^b^ | < 0.05 |
| NO_3_^−^-N (mg/L) | 0.43 ± 0.18^b^ | 0.23 ± 0.11^c^ | 0.20 ± 0.12^c^ | 0.60 ± 0.22^a^ | < 0.01 |
| NO_2_^−^-N (mg/L) | 0.023 ± 0.04 | 0.004 ± 0.00 | 0.001 ± 0.00 | 0.003 ± 0.00 | 0.089 |
| COD (mg/L) | 18.88 ± 7.47 | 12.25 ± 6.23 | 20.75 ± 7.95 | 21.63 ± 9.49 | 0.098 |
| Chl-a (μg/L) | 31.63 ± 20.09^b^ | 67.00 ± 26.18^a^ | 30.13 ± 6.79^bc^ | 11.75 ± 14.77^c^ | < 0.01 |
| OC (%) | 4.21 ± 2.99 | 4.98 ± 3.44 | 5.45 ± 3.85 | 6.53 ± 6.30 | 0.755 |
| STN (g/kg) | 1.19 ± 0.58 | 2.23 ± 1.20 | 2.80 ± 1.33 | 2.95 ± 2.68 | 0.155 |
| STP (g/kg) | 0.83 ± 0.12 | 0.79 ± 0.18 | 0.68 ± 0.19 | 0.79 ± 0.13 | 0.319 |
