# Supplementary material for: Comparing diversity patterns and processes of microbial community assembly in water column and sediment in Lake Wuchang, China
Source: PeerJ. 2023 Jan 5;11:e14592. doi: 10.7717/peerj.14592 (PMC9826614; doi:10.7717/peerj.14592)
Supplement: Supplemental Information 2 [file peerj-11-14592-s002.doc]

|  | water | | | | | | | | | | | | | | | sediment | | |
| --- | --- | --- | --- | --- | --- | --- | --- | --- | --- | --- | --- | --- | --- | --- | --- | --- | --- | --- |
|  | **Depth** | **WT** | **DO** | **pH** | **SD** | **EC** | **TSS** | **TP** | **P04-P** | **TN** | **NH4-N** | **NO3-N** | **NO2-N** | **COD** | **Chl-a** | OC | STN | STP |
| 6W1W | 1.6 | 22.6 | 7.51 | 7.4 | 51 | 119 | 7 | 0.11 | 0.08 | 1.9 | 0.55 | 0.8 | 0.081 | 3 | 7 | 2 | 832 | 755 |
| 6W2W | 1.8 | 24.7 | 8.02 | 8.33 | 52 | 146.9 | 8 | 0.093 | 0.04 | 0.8 | 0.09 | 0.5 | 0.003 | 18 | 41 | 2.3 | 994 | 938 |
| 6W3W | 1.7 | 24 | 7.6 | 7.98 | 95 | 135.8 | 6 | 0.093 | 0.05 | 1.3 | 0.33 | 0.2 | 0.005 | 19 | 16 | 1.4 | 642 | 656 |
| 6W4W | 1.7 | 24.9 | 8.49 | 8.24 | 50 | 140.6 | 9 | 0.097 | 0.05 | 1.1 | 0.1 | 0.3 | 0.003 | 17 | 42 | 2.2 | 1150 | 992 |
| 6W5W | 1.3 | 24.6 | 8.68 | 8.17 | 51 | 139.3 | 11 | 0.103 | 0.06 | 2.3 | 0.23 | 0.4 | 0.003 | 21 | 52 | 9.9 | 2090 | 906 |
| 6W6W | 1.5 | 24.5 | 10.08 | 8.84 | 57 | 150.1 | 11 | 0.103 | 0.03 | 1.5 | 0.19 | 0.5 | 0.002 | 24 | 61 | 5.4 | 1690 | 828 |
| 6W7W | 1.8 | 24.5 | 11.55 | 9.42 | 100 | 122.1 | 3 | 0.063 | 0.05 | 0.2 | 0.49 | 0.3 | 0.002 | 20 | 23 | 7 | 1710 | 708 |
| 6W8W | 2.9 | 24.6 | 2.71 | 7.44 | 35 | 187.1 | 13 | 0.203 | 0.14 | 4.2 | 2.29 | 0.4 | 0.081 | 29 | 11 | 3.5 | 443 | 813 |
| 8W1W | 5 | 30.3 | 6.61 | 7.97 | 110 | 103.1 | 7 | 0.153 | 0.02 | 1.1 | 0.11 | 0.1 | 0.002 | 8 | 64 | 3.3 | 1810 | 850 |
| 8W2W | 4.5 | 30.2 | 7.89 | 7.96 | 100 | 127 | 10 | 0.153 | 0.02 | 0.7 | 0.01 | 0.4 | 0.002 | 24 | 15 | 3.2 | 680 | 887 |
| 8W3W | 4.3 | 30.1 | 6.97 | 7.95 | 105 | 110.1 | 6 | 0.117 | 0.02 | 0.3 | 0.07 | 0.3 | 0.011 | 6 | 81 | 1.6 | 846 | 490 |
| 8W4W | 5 | 29.7 | 6.93 | 7.98 | 100 | 112 | 9 | 0.133 | 0.19 | 0.1 | 0.13 | 0.3 | 0.004 | 7 | 79 | 3.7 | 3240 | 1080 |
| 8W5W | 5.9 | 29.4 | 3.76 | 7.68 | 70 | 130.5 | 10 | 0.157 | 0.29 | 3.2 | 0.05 | 0.1 | 0.001 | 17 | 65 | 6.4 | 2200 | 632 |
| 8W6W | 4.8 | 29.7 | 6.19 | 7.78 | 80 | 131.3 | 8 | 0.143 | 0.04 | 0.4 | 0.02 | 0.3 | 0.004 | 8 | 97 | 12.1 | 4100 | 840 |
| 8W7W | 4.8 | 29.4 | 4.13 | 7.69 | 100 | 130 | 6 | 0.133 | 0.02 | 0.2 | 0.01 | 0.1 | 0.002 | 13 | 88 | 7.1 | 3200 | 811 |
| 8W8W | 2.8 | 30.3 | 1.96 | 7.37 | 80 | 120.8 | 10 | 0.147 | 0.04 | 0.1 | 0.01 | 0.2 | 0.002 | 15 | 47 | 2.4 | 1800 | 712 |
| 10W1W | 2.9 | 18.9 | 10.72 | 8.81 | 110 | 108.9 | 8 | 0.07 | 0.04 | 0.5 | 0.01 | 0.4 | 0.001 | 34 | 28 | 2.4 | 2140 | 748 |
| 10W2W | 3 | 18.4 | 9.47 | 8.43 | 115 | 109.4 | 9 | 0.063 | 0.04 | 0.4 | 0.01 | 0.1 | 0.001 | 19 | 32 | 2.9 | 2140 | 375 |
| 10W3W | 3.3 | 19 | 10.32 | 8.46 | 110 | 110.4 | 8 | 0.077 | 0.03 | 0.4 | 0.01 | 0.1 | 0.001 | 16 | 28 | 2 | 1730 | 712 |
| 10W4W | 3.1 | 18.8 | 9.24 | 8.4 | 100 | 110.8 | 11 | 0.07 | 0.02 | 0.3 | 0.01 | 0.1 | 0.001 | 25 | 25 | 3.3 | 1940 | 409 |
| 10W5W | 2.7 | 18.7 | 8.6 | 8.18 | 85 | 112.3 | 8 | 0.097 | 0.03 | 0.1 | 0.01 | 0.1 | 0.001 | 10 | 26 | 9.6 | 5380 | 854 |
| 10W6W | 2.6 | 18.5 | 9.7 | 8.36 | 60 | 127.3 | 11 | 0.087 | 0.03 | 0.1 | 0.01 | 0.3 | 0.003 | 18 | 33 | 10.1 | 3930 | 917 |
| 10W7W | 2.5 | 19.2 | 9.46 | 8.27 | 70 | 175.3 | 10 | 0.093 | 0.03 | 0.7 | 0.01 | 0.3 | 0.002 | 15 | 24 | 10.5 | 3450 | 716 |
| 10W8W | 2.7 | 18.9 | 2.03 | 7.83 | 140 | 165.7 | 6 | 0.107 | 0.04 | 0.2 | 0.01 | 0.2 | 0.001 | 29 | 45 | 2.8 | 1650 | 725 |
| 1W1W | 1.3 | 5.2 | 11.81 | 8.28 | 30 | 114 | 43 | 0.163 | 0.01 | 1.2 | 0.04 | 0.7 | 0.004 | 13 | 11 | 3.2 | 1240 | 634 |
| 1W2W | 1.5 | 5.1 | 12.23 | 8.26 | 25 | 127.7 | 72 | 0.173 | 0.01 | 0.6 | 0.07 | 0.8 | 0.005 | 12 | 10 | 3.2 | 1360 | 828 |
| 1W3W | 1.3 | 5.2 | 12.16 | 8.09 | 15 | 134.3 | 177 | 0.193 | 0.11 | 1.3 | 0.06 | 0.5 | 0.002 | 12 | 8 | 1.9 | 734 | 560 |
| 1W4W | 1.3 | 5.2 | 11.76 | 8.19 | 35 | 124.8 | 39 | 0.1 | 0.06 | 2.1 | 0.06 | 1 | 0.004 | 22 | 11 | 3.5 | 1420 | 912 |
| 1W5W | 1.7 | 9 | 10.94 | 8.32 | 65 | 153.9 | 7 | 0.097 | 0.03 | 2.7 | 0.01 | 0.4 | 0.002 | 26 | 3 | 9.6 | 4590 | 924 |
| 1W6W | 1.6 | 8.5 | 10.30 | 8.49 | 58 | 174.7 | 10 | 0.14 | 0.06 | 2.4 | 0.16 | 0.4 | 0.002 | 23 | 2 | 6.9 | 3700 | 786 |
| 1W7W | 1.3 | 8.8 | 10.67 | 8.33 | 53 | 206.2 | 12 | 0.067 | 0.03 | 1.3 | 0.22 | 0.4 | 0.003 | 40 | 2 | 20.8 | 8690 | 820 |
| 1W8W | 1.1 | 8.7 | 8.75 | 8.14 | 20 | 213 | 145 | 0.203 | 0.15 | 2.7 | 0.24 | 0.6 | 0.004 | 25 | 47 | 3.1 | 1850 | 842 |
